# Restorative Surgery in Adult Short Bowel Syndrome: Outcomes from a Single-Center Experience with an Illustrative Complex Case

**DOI:** 10.3390/jcm15082931

**Published:** 2026-04-12

**Authors:** Federica Galiandro, Carmen Nesci, Giulio Perrone, Franco Sacchetti, Angelo Eugenio Potenza, Dario Pastena, Sara Ennas, Marco Pizzoferrato, Franco Scaldaferri, Alfredo Papa, Luigi Sofo, Paola Caprino

**Affiliations:** 1 Abdominal Surgery Unit, Fondazione Policlinico Universitario “A. Gemelli”, IRCCS, Largo Agostino Gemelli, 8, 00136 Rome, Italy; carmennesci96@gmail.com (C.N.); drperronegiulio@gmail.com (G.P.); franco.sacchetti@policlinicogemelli.it (F.S.); angeloeugenio.potenza@policlinicogemelli.it (A.E.P.); dario.pastena@policlinicogemelli.it (D.P.); luigi.sofo@unicatt.it (L.S.); paola.caprino@policlinicogemelli.it (P.C.); 2 Internal Medicine and Gastroenterology Unit, Fondazione Policlinico Universitario “A. Gemelli”, IRCCS, Largo Agostino Gemelli, 8, 00136 Rome, Italy; sara.ennas@policlinicogemelli.it (S.E.); marco.pizzoferrato@policlinicogemelli.it (M.P.); franco.scaldaferri@unicatt.it (F.S.); 3 Centre for Digestive Diseases (CEMAD) and Gastroenterology Unit, Fondazione Policlinico Universitario “A. Gemelli”, IRCCS, Largo Agostino Gemelli, 8, 00136 Rome, Italy; alfredo.papa@policlinicogemelli.it

**Keywords:** short bowel syndrome, intestinal rehabilitation, restorative surgery, parenteral nutrition, hostile abdomen

## Abstract

**Background**: Short bowel syndrome (SBS) is a severe form of intestinal failure often associated with high output jejunostomy, fluid and electrolyte imbalance, and long-term dependence on parenteral nutrition (PN). In patients with type I SBS, restorative surgery may reduce PN dependence and enable conversion to type II or III SBS through restoration of intestinal continuity. **Methods**: We report our single-center experience. Between 2018 and 2025, nine adult patients with chronic type I SBS and high-output jejunostomy underwent restorative surgery within a multidisciplinary intestinal rehabilitation program. All patients were PN-dependent preoperatively, and two had intestinal failure-associated liver disease (IFALD). Surgical strategies were individualized according to residual anatomy and focused on restoration of intestinal continuity, without bowel lengthening procedures. Clinical outcomes were descriptively analyzed. **Results**: Intestinal continuity was successfully restored in all patients, resulting in conversion from type I to type II or III SBS. A clinically relevant improvement in intestinal function was observed in all cases, although follow-up duration was heterogeneous (range 3–60 months), with some patients still in early postoperative follow-up. Complete enteral autonomy was achieved in 3/9 patients (33%), while 6/9 patients (67%) experienced a reduction in PN requirements, including partial or nocturnal supplementation. Five of nine patients developed postoperative complications: one required reoperation and one endoscopic treatment for anastomotic bleeding. No 90-day postoperative mortality was recorded. **Conclusions**: In adult patients with type I SBS, restorative surgery enables anatomical and functional conversion to type II or III SBS. When performed within specialized multidisciplinary programs and guided by careful management of hostile abdomen, this approach may result in functional improvement and reduced PN dependence, although outcomes remain heterogeneous.

## 1. Introduction

Short bowel syndrome (SBS) is a rare but severe form of intestinal failure resulting from extensive small bowel resection, leading to inadequate nutrient and fluid absorption to sustain life without supplemental parenteral nutrition (PN) [1,2]. The most common etiologies include mesenteric ischemia, Crohn’s disease, postoperative complications, trauma, and congenital disorders [3]. Despite improvements in medical management and home PN programs, SBS remains a life-threatening condition associated with high morbidity, impaired quality of life, and a substantial healthcare burden [4]. Recent guidelines and clinical studies have further refined the multidisciplinary management of chronic intestinal failure, highlighting the role of integrated medical and surgical approaches [5,6].

The clinical severity of SBS depends on residual bowel length, the anatomical segment preserved, the presence of the colon in continuity, and the preservation of the ileocecal valve [7]. Short bowel length and rapid intestinal transit result in malabsorption, diarrhea, dehydration, electrolyte imbalance, and malnutrition. In this framework, type I SBS, characterized by an end jejunostomy without intestinal continuity, represents the most severe form in terms of fluid loss and metabolic instability, whereas type II and type III SBS are associated with progressively improved absorptive capacity due to partial or complete restoration of intestinal continuity and colonic integration [1,2,7] The classification of short bowel syndrome into type I (end jejunostomy), type II (jejuno-colic anastomosis), and type III (jejuno-ileal anastomosis with colon in continuity) was based on the ESPEN (The European Society for Clinical Nutrition and Metabolism) definition of intestinal failure, widely used in adult clinical practice [8]. Following resection, the intestine undergoes an adaptive process characterized by mucosal hyperplasia, bowel dilation, and changes in motility and transporter expression; this response may be enhanced by enteral feeding, hormonal factors such as glucagon-like peptide-2 (GLP-2), and surgical strategies that optimize bowel geometry and transit time [9,10].

Within this context, restorative surgery represents a cornerstone of intestinal rehabilitation programs, aiming to increase functional absorptive capacity, reduce intestinal transit, improve nutrient absorption, and minimize long-term PN-related complications such as intestinal failure-associated liver disease (IFALD) and catheter-related infections [11]. Surgical intervention is generally considered after completion of the adaptive phase and stabilization under optimized medical therapy [12]. A major and clinically relevant objective of restorative surgery in SBS is conversion from type I SBS, characterized by high-output jejunostomy, to type II or III SBS through restoration of intestinal continuity whenever excluded bowel segments are available. By re-establishing intestinal continuity and, when feasible, colonic integration, restorative surgery may fundamentally modify the anatomical and functional classification of SBS, thereby improving intestinal absorption and facilitating nutritional rehabilitation [7,13,14].

Several reconstructive strategies have been described as part of autologous gastrointestinal reconstructive surgery (AGIR), including bowel lengthening procedures such as longitudinal intestinal lengthening and tailoring (LILT) and serial transverse enteroplasty (STEP), anti-peristaltic reversed segments, restoration of intestinal continuity, and, more recently, biomimetic experimental approaches such as the Saeki–Spiral–Shark (3S) technique [15,16]. Although several autologous gastrointestinal reconstructive procedures have been described, no bowel lengthening techniques were performed in the present cohort, as further discussed below. Intestinal transplantation remains an option for patients with irreversible intestinal failure and life-threatening PN-related complications after failure of rehabilitative strategies, although it is associated with substantial morbidity [17,18]. In our cohort, no patient required or proceeded to intestinal transplantation during the study period. Overall, restorative procedures have been shown to reduce PN dependence and improve quality of life in selected patients, but they remain technically demanding and may be associated with complications such as anastomotic leaks, bowel ischemia, bacterial overgrowth, and redilatation [5,19,20]. In adult patients, these operations are often particularly challenging because of multiple previous abdominal procedures, peritonitis, adhesions, and hostile abdomen.

In this study, we report our recent single-center clinical experience with restorative surgery in adult patients with type I SBS, with particular attention to functional outcomes and to a particularly complex illustrative case.

## 2. Materials and Methods

### 2.1. Study Design and Patient Selection

We retrospectively analyzed clinical outcomes of adult patients with SBS and intestinal failure managed at our center. During the study period, approximately 40 patients with SBS were followed within our multidisciplinary intestinal rehabilitation program. Among this population, a subgroup of patients who underwent restorative surgery was identified and included in the present analysis, representing a minority of the overall cohort.

All patients presented preoperatively with type I SBS, characterized by a high output jejunostomy and complete dependence on PN. Patients were considered candidates for restorative surgery after completion of the intestinal adaptive phase and optimization of medical and nutritional therapy. Completion of the adaptive phase was defined based on clinical stabilization, including stable nutritional requirements, absence of ongoing sepsis, and no major changes in intestinal output over time. Favorable residual anatomy was defined by the presence of viable and well-vascularized bowel segments, absence of active intra-abdominal infection, and availability of reconstructible intestinal segments allowing restoration of continuity. Enteral autonomy was defined as complete independence from parenteral nutrition for at least 3 consecutive months. Reduction in parenteral nutrition was defined as a decrease in PN volume or frequency compared to baseline, including transition to partial or nocturnal supplementation.

Clinically meaningful improvement was defined as a composite outcome including improved hydration status, decreased PN requirements, and advancement of enteral intake, as assessed during follow-up. Exclusion criteria included ongoing intra-abdominal sepsis, severe clinical instability, and conditions precluding major abdominal surgery.

### 2.2. Preoperative Work-Up

In complex cases of SBS, preoperative work-up plays a pivotal role in guiding restorative surgery. Before any surgical intervention, it is essential to obtain the most accurate and detailed understanding possible of the patient’s residual anatomy. This includes assessment of the remaining bowel length, gastrointestinal continuity, presence and configuration of anastomoses, potential strictures, excluded segments, and spatial relationships between intestinal loops. Residual bowel length was determined based on intraoperative measurements when available, supplemented by review of prior operative reports and imaging studies.

Radiological evaluations were central to preoperative assessment. Upper gastrointestinal contrast studies provided valuable morphological and functional information on the gastroduodenal tract and proximal small bowel, allowing identification of delayed transit, areas of stasis, dilatation, or clinically relevant strictures. Cross-sectional imaging was also used to define residual anatomy and to support surgical planning, thereby minimizing intraoperative uncertainty during restorative procedures.

### 2.3. Surgical Strategy

Restorative surgical procedures were individualized according to anatomical and functional characteristics, with restoration of intestinal continuity representing the primary surgical objective. Tailored reconstructive strategies included jejuno-colic, jejuno-ileal, jejuno-jejunal, gastro-jejunal, and ileo-colic anastomoses, performed alone or in combination depending on residual bowel configuration.

### 2.4. Postoperative Management and Outcomes

The primary outcome was restoration of intestinal continuity with conversion from type I to type II or III SBS. Secondary outcomes included postoperative parenteral nutrition requirements, achievement of enteral autonomy, and postoperative morbidity and mortality. Enteral autonomy was defined as complete independence from parenteral nutrition for at least three consecutive months. Reduction in parenteral nutrition was defined as a decrease in volume or frequency compared to baseline, including transition to partial or nocturnal supplementation. Clinically meaningful improvement was defined as a composite outcome including reduction in parenteral nutrition requirements, improved hydration status, and advancement of enteral intake. Postoperative complications were retrospectively classified according to the Clavien–Dindo classification system [21].

Parenteral nutrition tapering was guided by clinical parameters including oral or enteral intake, hydration status, and biochemical markers such as electrolytes and renal function.

Postoperative care was delivered within a specialized intestinal failure program in close collaboration with nutrition specialists. Early enteral feeding was initiated when tolerated, followed by gradual tapering of PN and strict monitoring of fluid and electrolyte balance. Patients underwent regular clinical, nutritional, and biochemical follow-up.

### 2.5. Statistical Analysis

Given the limited sample size, data were analyzed descriptively. Continuous variables are reported as medians and ranges, while categorical variables are presented as absolute numbers and percentages. No inferential statistical analysis was performed.

### 2.6. Institutional Review Board

This study was conducted in accordance with the Declaration of Helsinki and approved by the Institutional Review Board (IRB No. 5483). All patients provided informed consent for data collection and publication.

## 3. Results

### 3.1. Clinical Outcomes

Between 2018 and 2025, nine adult patients with chronic SBS were managed at our institution within a dedicated multidisciplinary intestinal rehabilitation program. All patients presented with intestinal failure secondary to extensive small bowel resection due to heterogeneous etiologies, including intestinal ischemia, Crohn’s disease, abdominal trauma, visceral myopathy/intestinal pseudo-obstruction, diverticular disease, and ileal volvulus complicated by duodenal stenosis. Detailed patient characteristics are summarized in Table A1 (Appendix A).

All patients were dependent on PN preoperatively and presented with type I SBS.

No bowel lengthening procedures, including STEP, were performed in this cohort. Additional reconstructive maneuvers were guided by intraoperative findings and vascular assessment. Surgical timing was individualized, with restorative procedures undertaken after a median interval of approximately 11 months (range 6–56 months) from the last intestinal resection, aiming to balance intestinal adaptation with operative safety. Surgical and clinical outcomes are summarized in Table A2 (Appendix A).

Following restorative surgery and anatomical conversion to type II or type III SBS, a clinically relevant improvement in intestinal function was observed across the cohort. Complete enteral autonomy was achieved in 3/9 patients (33%), while 6/9 patients (67%) experienced a reduction in PN requirements.

Intestinal continuity was successfully restored in all patients, resulting in conversion from type I SBS to type II or type III SBS according to the final anatomical configuration.

A 22% (2/9) rate of severe postoperative complications (Clavien–Dindo grade ≥ III) was observed, as detailed in Table A2. No 90-day postoperative mortality was observed.

Follow-up duration ranged from 3 to 60 months. Patients with shorter follow-up (Cases 7 and 8) were still in the early postoperative phase, and their PN dependence status should be interpreted accordingly. Overall, restorative surgery was well tolerated and resulted in improved nutritional status and intestinal function in most patients.

### 3.2. Illustrative Complex Case: Patient 6

We report a particularly complex case of SBS that could not be classified within existing anatomical categories. A 59-year-old male presented to our center in June 2025 with a diverting jejunostomy located approximately 30 cm distal to the ligament of Treitz, stenosis of the third portion of the duodenum managed with a duodenal drainage catheter, and a mucous fistula of the terminal ileum (40 cm) with the colon preserved (type I SBS with excluded distal bowel and closed duodenum). These findings were the consequence of multiple previous abdominal surgeries performed for desmoid-type fibromatosis of the small bowel.

### 3.3. Clinical History

The patient’s clinical history began in October 2021 with identification of a large mesenteric mass involving the ileal mesentery and the root of the mesentery. Histopathology confirmed desmoid-type fibromatosis with CTNNB1 (Beta-1 Catenin Syndrome) exon 3 mutation. Initial management consisted of radiological surveillance followed by systemic chemotherapy with methotrexate and vinorelbine, resulting in partial radiological response and subsequent disease stabilization.

In February 2024, the patient developed intestinal obstruction requiring emergency surgery with resection of approximately one meter of ileum and ileo-ileal anastomosis, which was complicated by anastomotic leakage. Subsequent procedures included resection of the failed anastomosis with creation of a jejunostomy and ileal mucous fistula, repair of a perforation of the third portion of the duodenum with placement of a prepapillary drainage catheter, and multiple reoperations for duodeno-cutaneous fistula managed with an open abdomen technique. Following discharge, the patient was treated at an intestinal failure center with total parenteral nutrition.

### 3.4. Preoperative Evaluation

**Figure 1:**
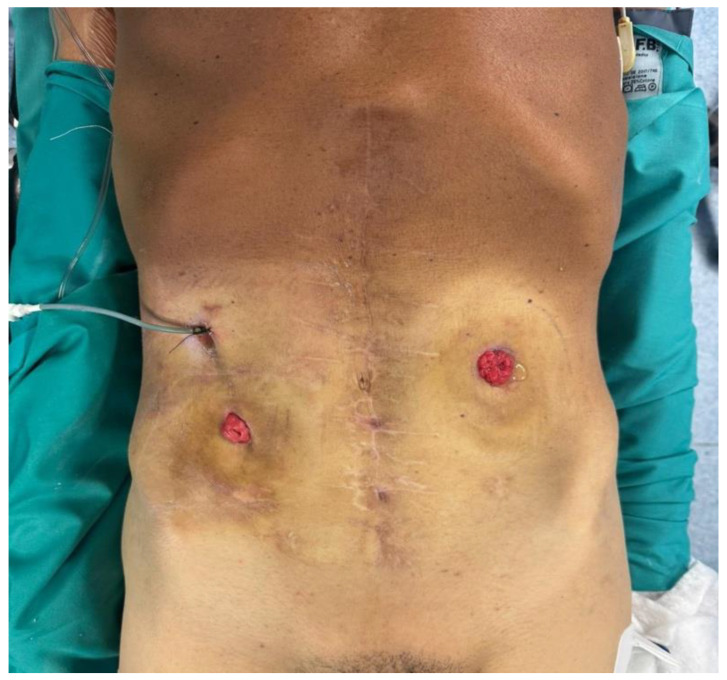
Preoperative abdominal photograph showing a left-sided jejunostomy and a duodenal drain in situ, with a mucous fistula in the right flank.

**Figure 2:**
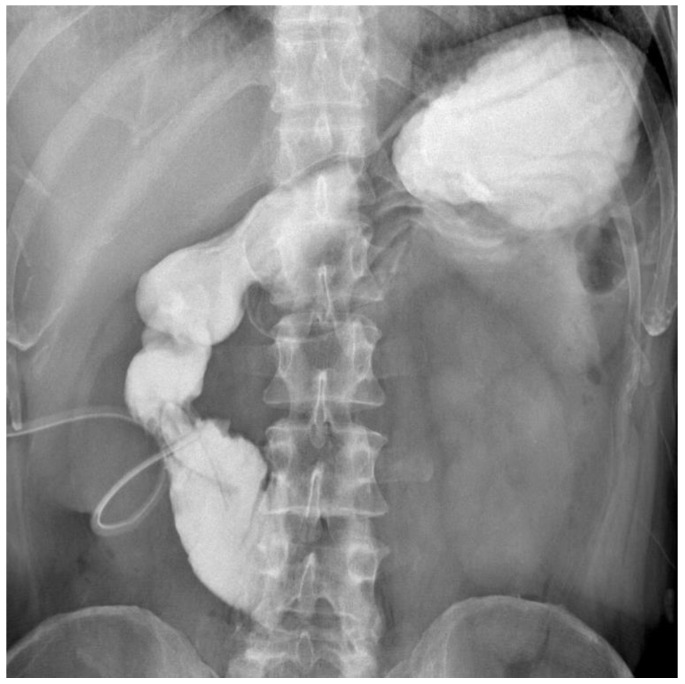
Preoperative upper GI contrast study with oral contrast shows an obstruction of the third portion of the duodenum. A duodenal drain is in place.

**Figure 3:**
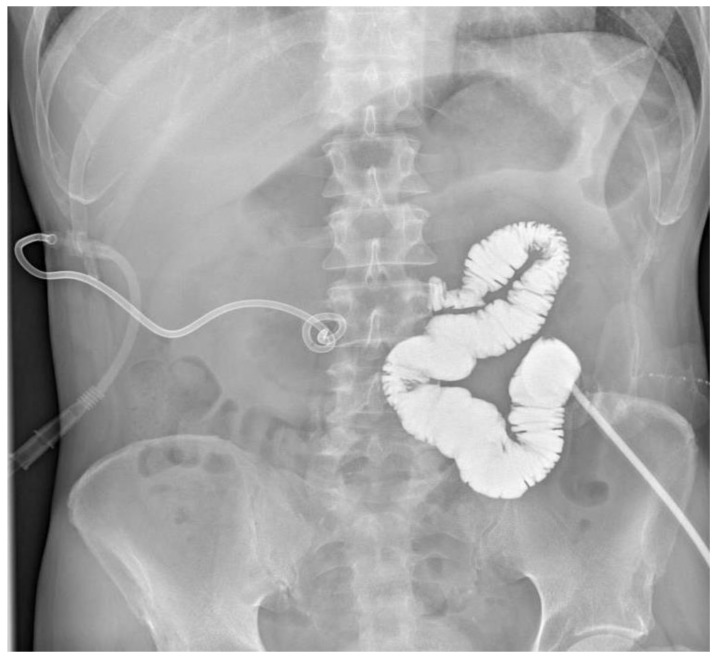
Contrast opacification through the jejunostomy showing a proximal stop at the level of the third portion of the duodenum (30 cm of jejunum).

**Figure 4:**
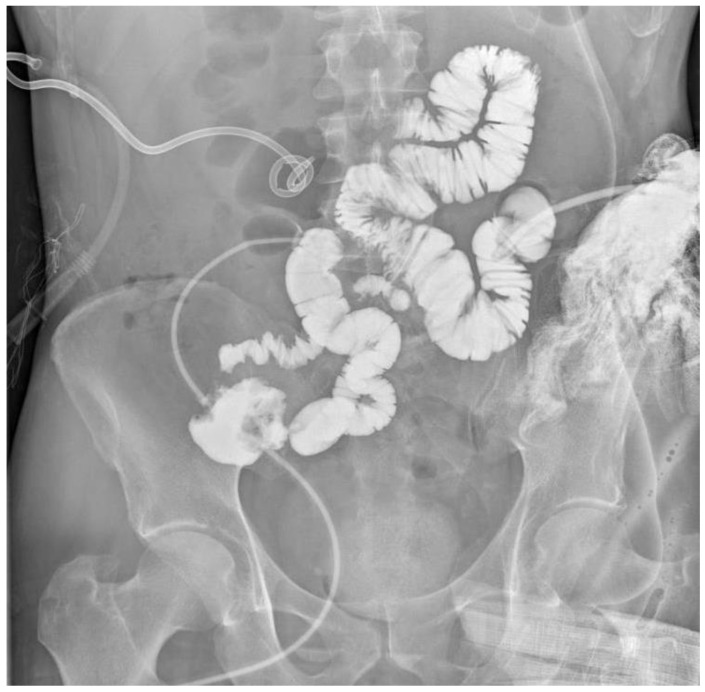
Following the study described in Figure 3, contrast medium was administered through the ileal mucous fistula, allowing identification of the remaining small bowel up to the ileocecal valve (40 cm).

**Figure 5:**
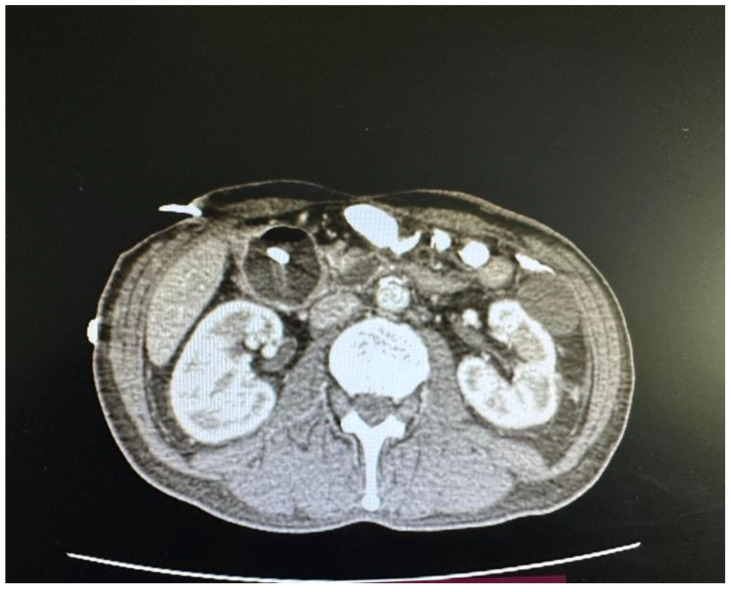
Axial abdominal CT image reports demonstrating dilatation with a duodenal drain in situ.

**Figure 6:**
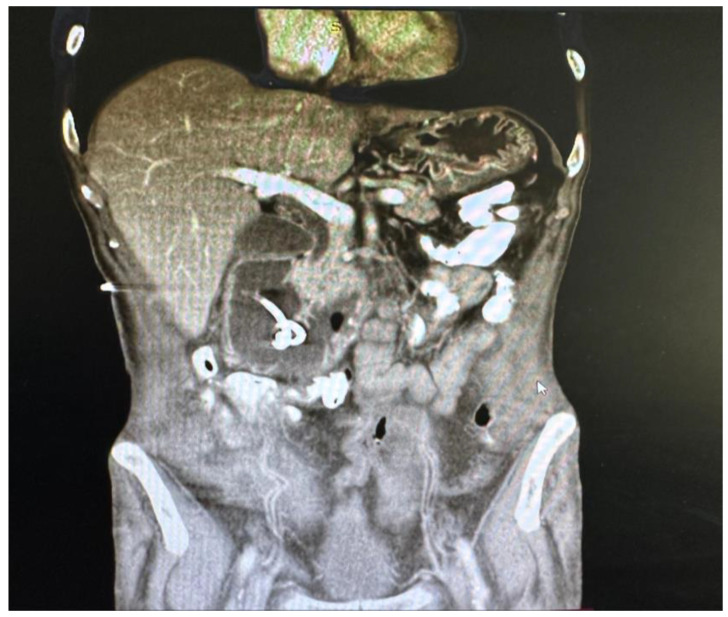
Coronal abdominal CT image demonstrating duodenal stenosis with a duodenal drain in situ.

In March 2025, the patient was admitted to our institution for evaluation of potential intestinal reconstruction (Figure 1). Endoscopy confirmed a tight stenosis of the third portion of the duodenum. Contrast studies demonstrated limited jejunal continuity proximal to the stoma and preserved distal ileal segments reaching the cecum. CT imaging showed postoperative changes without clear signs of oncological disease recurrence (Figure 2, Figure 3, Figure 4, Figure 5 and Figure 6).

### 3.5. Surgery

**Figure 7:**
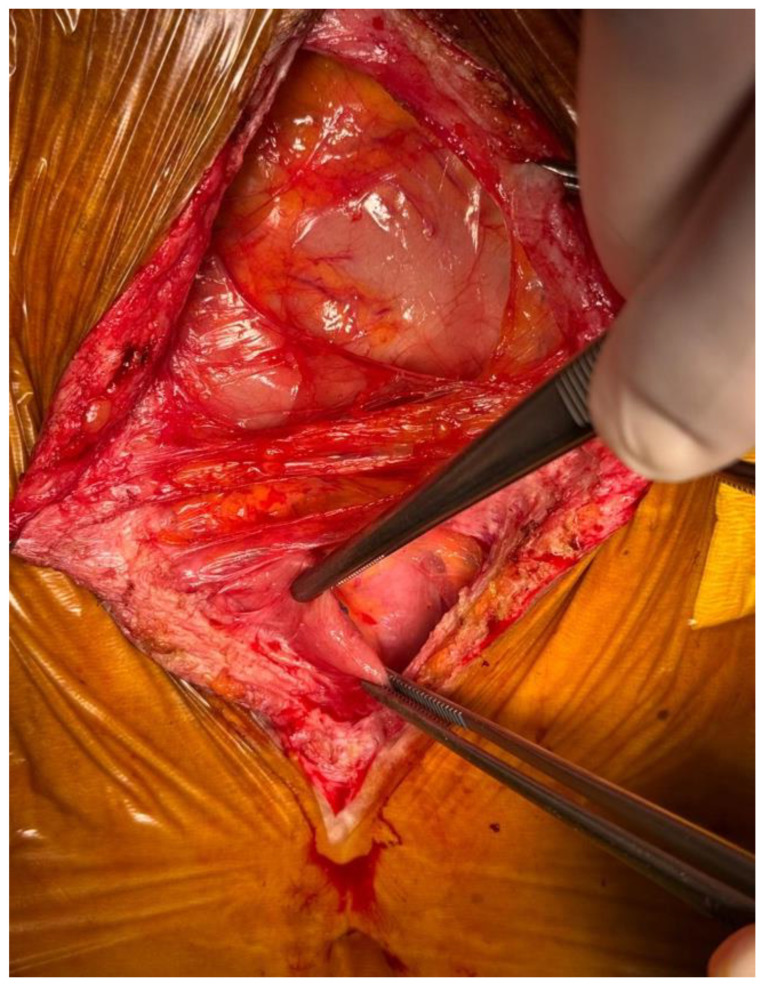
Dense visceroparietal and viscerovisceral adhesions with a hostile abdomen.

**Figure 8:**
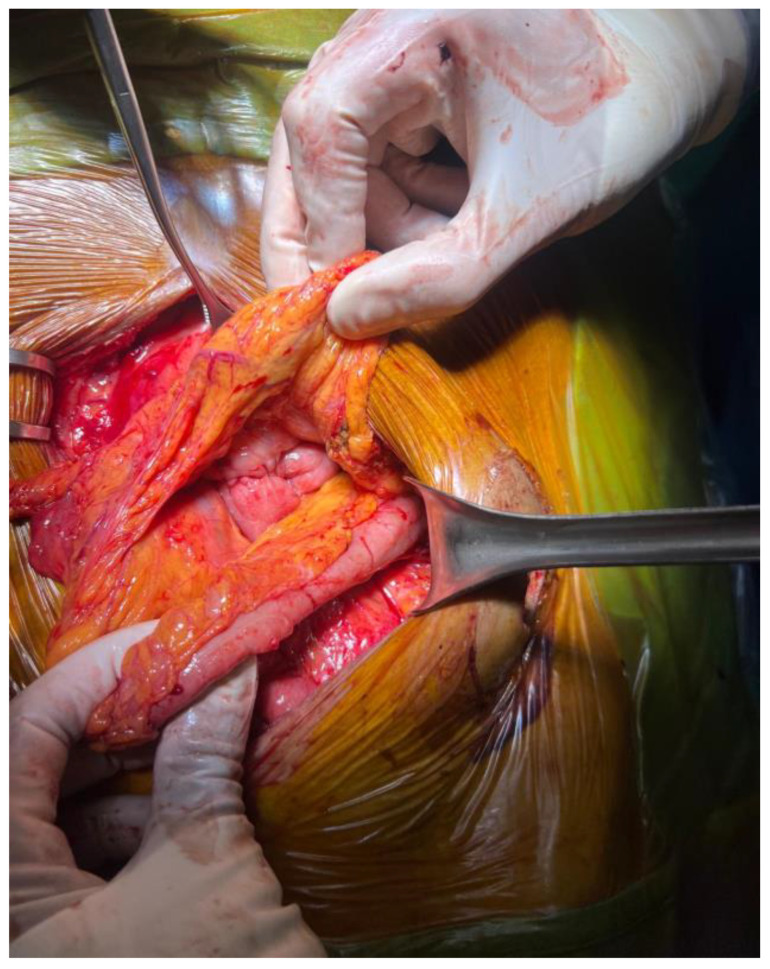
Gastroenteric anastomosis on the posterior wall of the stomach with a transmesocolic loop.

**Figure 9:**
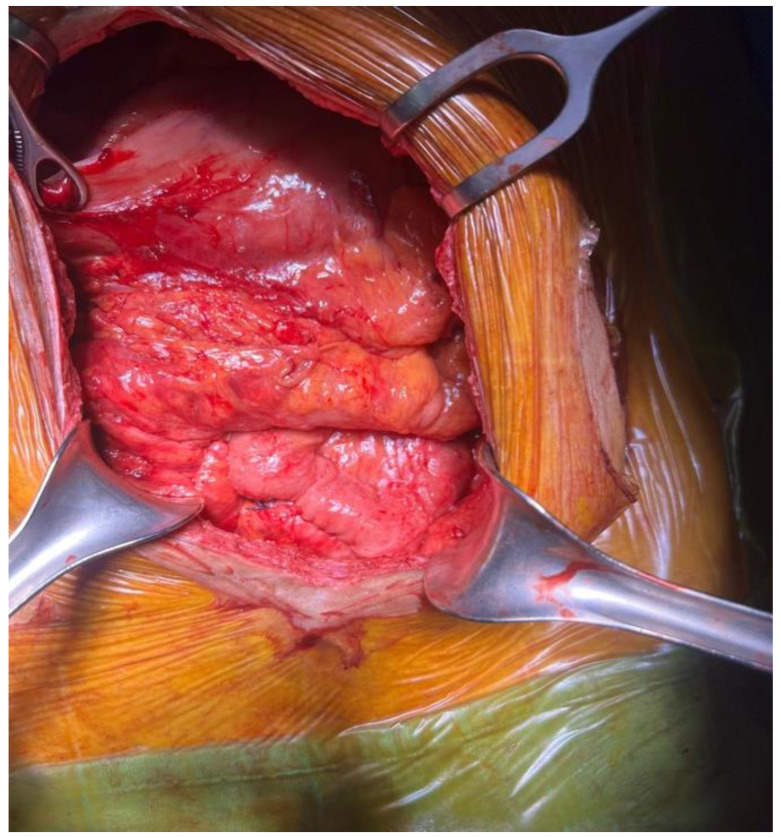
Side-to-side jejunoileal anastomosis.

Reconstructive surgery was performed in June 2025. Exploratory laparotomy revealed extensive intra-abdominal adhesions requiring careful adhesiolysis (Figure 7). The jejunostomy and ileal mucous fistula were mobilized. Reconstruction was achieved through a transmesocolic mechanical gastrojejunostomy and a side-to-side isoperistaltic jejunoileal anastomosis after confirmation of adequate bowel perfusion (Figure 8 and Figure 9). Abdominal drains were placed near both anastomoses.

### 3.6. Postoperative Course

The postoperative course was characterized by delayed gastric emptying and delayed functional activation of the gastroenteric anastomosis, likely related to preferential drainage of gastric, pancreatic, and biliary secretions through the indwelling intraduodenal drainage catheter, which diverted digestive fluids away from the newly created anastomosis.

To manage this condition, an endoscopically placed nasogastric tube was inserted to decompress the stomach and reduce the risk of aspiration pneumonia, while a nasojejunal feeding tube was positioned distal to the anastomosis for enteral nutritional support.

After approximately one month, the patient was discharged with the duodenal drainage catheter left in situ (kept closed) and receiving parenteral nutrition and enteral nutrition through a nasojejunal tube. The nasogastric tube was maintained in place.

**Figure 10:**
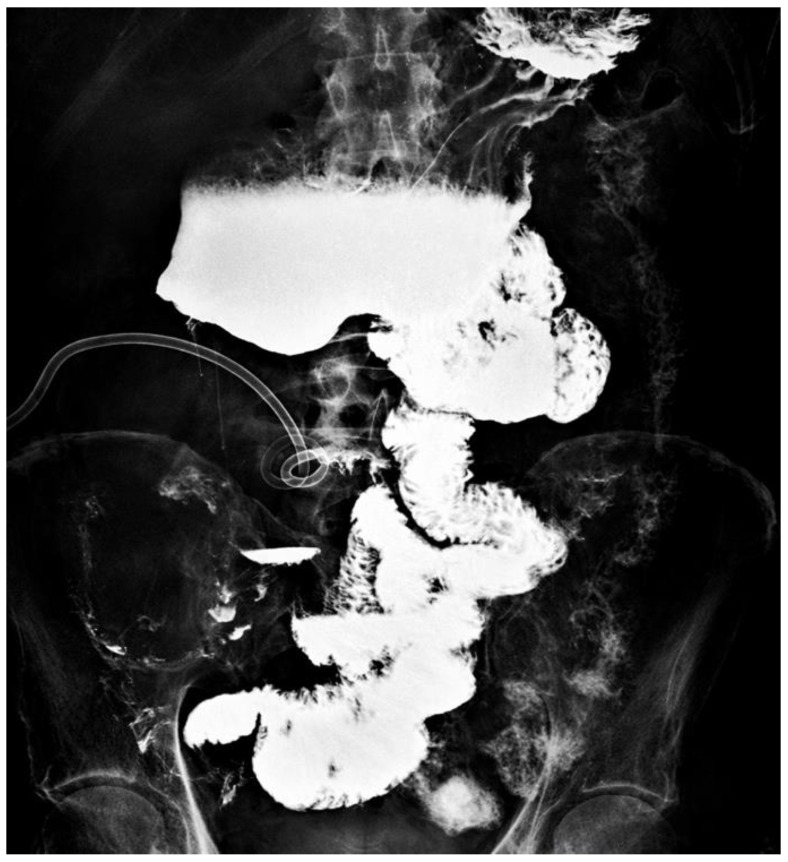
Contrast-enhanced radiographic study (oral contrast) performed 3 months after surgery showing normal patency of the gastroenteroanastomosis, with no evidence of duodenal stasis.

**Figure 11:**
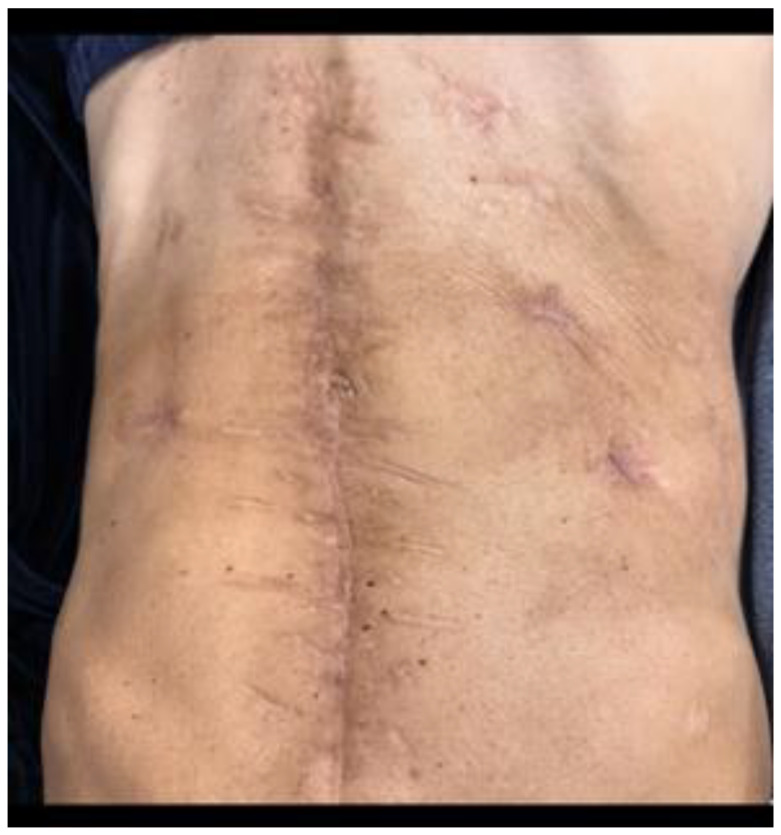
Postoperative abdominal appearance of the patient.

In November 2025, a plain abdominal radiograph demonstrated functional recanalization of the gastroenteric anastomosis (Figure 10). Consequently, once the nasogastric tube stopped draining and the patient was able to feed regularly with the tubes in place, both the nasogastric and nasojejunal tubes were removed. One month after the abdominal radiograph, the duodenal drainage catheter was also removed. The patient continued parenteral nutrition with progressive weaning while demonstrating tolerance to total enteral feeding (Figure 11).

The favorable restoration of anastomotic transit was likely facilitated by pyloric hypotonia secondary to chronic duodenal obstruction. The resulting pyloric dilation and incompetence may have promoted retrograde reflux of duodenal contents into the stomach, thereby facilitating passage through the gastroenteric anastomosis.

This illustrative case emphasizes several key lessons: the importance of detailed preoperative anatomical assessment, the need for flexible intraoperative decision-making, and the feasibility of achieving functional reconstruction even in highly complex anatomical settings.

Overall, the remaining patients presented heterogeneous clinical histories characterized by multiple prior abdominal surgeries, recurrent postoperative complications, and prolonged dependence on parenteral nutrition. The most common underlying conditions included mesenteric ischemia, Crohn’s disease, abdominal trauma, and functional disorders such as visceral myopathy.

Preoperatively, all patients exhibited high-output jejunostomy with significant fluid and electrolyte imbalance requiring long-term parenteral support. The number of previous abdominal procedures ranged from 2 to 5, frequently contributing to complex intra-abdominal adhesions and hostile abdomen conditions.

Following restorative surgery, most patients demonstrated progressive improvement in intestinal function, with improved hydration status, and gradual advancement of enteral intake. The degree of parenteral nutrition reduction varied according to residual bowel length, presence of colon in continuity, and underlying motility disorders, as detailed in Table A2.

## 4. Discussion

In adult patients with type I SBS, restoration of intestinal continuity represents a key therapeutic objective that may significantly alter intestinal physiology and clinical trajectory. Conversion to type II or type III SBS enables improved fluid and electrolyte absorption, enhanced nutrient salvage, and reduced dependence on parenteral nutrition. While anatomical conversion was achieved in all patients, functional outcomes varied considerably. Patients achieving type III configuration generally demonstrated greater functional recovery and a higher likelihood of enteral autonomy compared to those with type II anatomy.

A distinctive feature of our series is that all patients initially presented with type I SBS, characterized by a high-output jejunostomy, a condition associated with profound fluid and electrolyte losses and a particularly high burden of PN dependence. In this context, restorative surgery was not merely aimed at reducing PN requirements, but at achieving a fundamental anatomical and functional conversion to type II or type III SBS through restoration of intestinal continuity. This transition represents a critical turning point in the natural history of SBS, as it substantially modifies intestinal physiology, absorptive capacity, and metabolic stability [7,13,14].

A key component of our surgical approach was the systematic restoration of intestinal continuity whenever anatomically feasible. This strategy is well supported by the literature, which consistently demonstrates that the presence of the colon in continuity plays a pivotal role in enhancing fluid and electrolyte absorption and in salvaging energy through colonic fermentation of carbohydrates into short-chain fatty acids [7,13]. In this context, the reduction in PN requirements observed in most of our patients appears coherent with established physiological principles and prior clinical studies showing that colonic reintegration is among the strongest predictors of PN weaning in SBS [14]. Conversely, the variability in achieving complete enteral autonomy likely reflects interindividual differences in residual small bowel length, preservation of the ileocecal valve, baseline motility, and the presence of associated upper gastrointestinal pathology, all factors known to influence long-term functional outcomes [2].

Notably, no bowel lengthening procedures, including STEP, were performed in our cohort. While STEP has been shown to be effective in selected patients, particularly in the presence of markedly dilated small bowel and favorable mesenteric anatomy, its role in adult SBS remains more selective than in pediatric populations [20,22]. The absence of bowel lengthening procedures in this cohort was primarily related to the lack of significant bowel dilation and, in some cases, unfavorable mesenteric anatomy or complex prior surgical history. Our experience suggests that meaningful functional improvement can still be achieved through anatomy-driven restorative procedures focused on continuity restoration, without bowel lengthening, especially in patients with complex surgical histories. In several cases, persistent partial PN dependence after reconstruction can be reasonably explained by factors frequently reported in the literature, such as extremely short residual bowel length, impaired motility disorders including visceral myopathy or intestinal pseudo-obstruction, or proximal disease such as duodenal stenosis, which may limit nutrient delivery and absorption despite technically successful anastomoses [10].

From a safety standpoint, postoperative morbidity in our series was acceptable and manageable within a specialized intestinal failure setting, with no perioperative mortality observed. This is particularly relevant given that many patients with SBS undergoing restorative surgery present with a hostile abdomen, characterized by dense adhesions, distorted anatomy, friable bowel, prior septic events, fistulae, and multiple previous laparotomies. Lundy and Ashburn emphasize that surgery in the hostile abdomen represents one of the most challenging scenarios in abdominal surgery and requires individualized goal setting, careful preoperative optimization, thoughtful timing, and an experience-driven intraoperative strategy focused on safety and preservation of remaining bowel length [23].

In our practice, adherence to hostile abdomen management principles, including multidisciplinary preoperative optimization, correction of nutritional and metabolic derangements, control of sepsis, and delayed re-entry after the last major resection, likely contributed to the favorable safety profile observed. Furthermore, intraoperative strategies aimed at preserving bowel length, avoiding unnecessary resections, and maintaining a clear exit strategy, as advocated in hostile abdomen surgery, were integral to our operative planning and execution. In patients with hostile abdomen, surgery was performed after careful timing and progressive adhesiolysis, prioritizing bowel preservation and minimizing the risk of enterotomy [24,25].

An important consideration emerging from our experience is the definition of success in adult SBS. While complete independence from PN represents an ideal endpoint, a sustained reduction in PN volume or frequency, often allowing transition to partial or nocturnal PN, may represent a clinically meaningful and patient-centered outcome. Such improvements are associated with reduced catheter-related complications, lower risk of IFALD, and improved quality of life [4,5,11]. This perspective is increasingly reflected in contemporary intestinal rehabilitation programs, where incremental functional gains are recognized as valuable outcomes, particularly in patients with complex anatomy or hostile abdomen features [12,19]. No patients in this cohort received GLP-2 analogue therapy (e.g., teduglutide), reflecting local availability and treatment policies during the study period. The rates of complete enteral autonomy (33%) and postoperative complications (55%) observed in our cohort are broadly consistent with previously reported adult SBS series, in which enteral autonomy rates typically range between 20% and 50%, depending on residual anatomy and duration of follow-up [4,14].

The limitations of this study include its retrospective nature, small sample size, and heterogeneity in underlying etiologies and reconstructive configurations. Nonetheless, the real-world characteristics of the cohort and the consistency of outcomes with established physiological and surgical principles support the role of restorative surgery as a key component of multidisciplinary intestinal rehabilitation in carefully selected adult patients. The lack of standardized quality-of-life assessment represents an additional limitation. Future multicenter studies and prospective registries may help refine patient selection criteria, optimize timing of reconstruction, and better define long-term outcomes, particularly in the challenging subset of patients with hostile abdomen anatomy.

### Future Directions

Emerging strategies in the management of SBS include combined medical-surgical approaches integrating optimized nutritional support, pharmacological therapies such as GLP-2 analogues, and individualized surgical reconstruction. Recent evidence and updated ESPEN guidelines emphasize the central role of multidisciplinary intestinal rehabilitation programs in improving intestinal adaptation, reducing parenteral nutrition dependence, and enhancing long-term outcomes in adult patients with chronic intestinal failure [1,23].

## 5. Conclusions

SBS is a complex condition requiring an integrated, multidisciplinary approach. Restorative surgery plays a pivotal role in intestinal rehabilitation by enhancing adaptation and reducing reliance on PN. In patients with type I SBS, conversion to type II or III SBS through restoration of intestinal continuity represents a fundamental anatomical and functional milestone, enabling improved metabolic stability and meaningful clinical recovery. Complete enteral autonomy was achieved in a subset of patients, while others required ongoing partial parenteral nutrition support, particularly in the context of limited follow-up duration. Continued innovation in surgical techniques and adjunctive therapies is essential to further improve long-term outcomes. Future research should focus on prospective multicenter studies with standardized outcome measures, improved characterization of the intestinal adaptive phase, and integration of surgical and pharmacological therapies such as GLP-2 analogues.

## Data Availability

The original contributions presented in this study are included in the article. Further inquiries can be directed to the corresponding author.

## Abbreviations

The following abbreviations are used in this manuscript:

SBS	Short Bowel Syndrome
PN	Parental Nutrition
IFALD	Intestinal Failure-Associated Liver Disease
ESPEN	The European Society for Clinical Nutrition and Metabolism
GLP-2	Glucagon-like peptide-2
AGIR	Autologous GastroIntestinal Reconstructive surgery
LILT	Longitudinal Intestinal Lengthening and Tailoring
STEP	Serial Transverse Enteroplasty
3S	Saeki-Spiral-Shark technique
CTNNB1	Beta-1 Catenin Syndrome

## Appendix A

**Table A1:** Clinical characteristics of patients undergoing restorative surgery for short bowel syndrome (SBS).

	Case 1	Case 2	Case 3	Case 4	Case 5	Case 6	Case 7	Case 8	Case 9
Age at restorative surgery (years)	47	59	37	44	42	59	64	56	64
Sex	F	F	F	M	M	M	M	M	M
Etiology of short bowel syndrome	intestinal ischemia, multiple resections, postoperative complications	Abdominal trauma, postoperative complications	Visceral myopathy/intestinal pseudo-obstruction, multiple resections, postoperative complications	Diverticular abscess, multiple resections (subtotal colectomies and jejuno-ileal resections), postoperative complications	Crohn’ disease, multiple resections, postoperative complications	multiple intestinal resections following ileal volvulus complicated by duodenal stenosis	Crohn’ disease, multiple resections, postoperative complications	intestinal volvolus, multiple resections, postoperative complications	Crohn’ disease, multiple resections, postoperative complications
Previous surgery (n)	5	2	3	5	2	4	4	3	2
SBS Type pre-surgery	1(J)	1 (J), IFALD	1 (J)	1 (J)	1 (J)	1 (J) *	1 (J), IFALD	1 (J)	1 (J)
Residual small bowel length in continuity (cm)	75	50	70	100	35	30 (with duodenal stenosis) jejunal + 45 ileum	100	140	150
Colon preserved (Yes/No)	Yes	Yes	Yes	No	Yes	Yes	Yes	Yes	Yes
Ileocecal valve preserved (Yes/No)	No	Yes	No	No (J-rectal anastomosis)	No	Yes	Yes	Yes	Yes
Time from last resection to restorative surgery (months)	11	10	6	13	6	17	56	24	12
Preoperative PN support	Yes	Yes	Yes	Yes	Yes	Yes	Yes	Yes	Yes

* Terminal duodenal fibrotic obstruction with about 30 cms of inhabited jejunal tract. PN: Parenteral Nutrition; IFALD: Intestinal Failure-Associated Liver Disease.

**Table A2:** Outcomes of patients undergoing restorative surgery for short bowel syndrome (SBS).

	Case 1	Case 2	Case 3	Case 4	Case 5	Case 6	Case 7	Case 8	Case 9
Type of restorative procedure	J-C anastomosis	J-J anastomosis, J-I anastomosis	J-C anastomosis	J-J anastomosis	J-I anastomosis, I-C anastomosis	G-J anastomosis, J-I anastomosis	J-I anastomosis	J-I anastomosis	J-I anastomosis
STEP (AGIR) performed (Yes/No)	No	No	No	No	No	No	No	No	No
Restoration of intestinal continuity (Yes/No)	Yes	Yes	Yes	Yes	Yes	Yes	Yes	Yes	Yes
Final small bowel length in continuity (cm)	75	80	70	140	115	75	110	155	180
Estimated residual colon in continuity (%)	47%	100%	60%	0%	60%	100%	100%	100%	100%
SBS Type post-surgery	II (JC)	III (JIC)	II (JC)	II (JR)	II (JC)	III (JIC)	III (JIC)	III (JIC)	III (JIC)
Postoperative PN requirement	Yes	Yes	Yes	Yes	Yes	Yes	Yes	Yes	No
Weaning off parental nutrition (Yes/No)	partial	partial	partial	Yes	Yes	Yes	No	No	partial
Postoperative complications	None	Post-operative Ileus, Urinary sepsis, anemia	None	None	Bleeding (endoscopic treatment)	Post-operative Ileus	Reoperation (bleeding, anastomotic leakage)	Post-operative Ileus, intra-abdominal abscess	None
Length of hospital stay (days)	7	30	10	8	13	36	49	25	6
Follow-up duration (months)	60	30	20	14	58	8	4	3	3

STEP: Serial Transverse Enteroplasty; AGIR: Autologous GastroIntestinal Reconstructive surgery.
